# Stromal protein degradation is incomplete in *Arabidopsis thaliana* autophagy mutants undergoing natural senescence

**DOI:** 10.1186/1756-0500-6-17

**Published:** 2013-01-17

**Authors:** Travis A Lee, Scott W Vande Wetering, Judy A Brusslan

**Affiliations:** 1 Department of Biological Sciences, California State University, Long Beach, 1250 Bellflower Boulevard, Long Beach, CA 90840-9502, USA

**Keywords:** Autophagy, Leaf senescence, Stromal protein degradation

## Abstract

**Background:**

Degradation of highly abundant stromal proteins plays an important role in the nitrogen economy of the plant during senescence. Lines of evidence supporting proteolysis within the chloroplast and outside the chloroplast have been reported. Two extra-plastidic degradation pathways, chlorophagy and Rubisco Containing Bodies, rely on cytoplasmic autophagy.

**Results:**

In this work, levels of three stromal proteins (Rubisco large subunit, chloroplast glutamine synthetase and Rubisco activase) and one thylakoid protein (the major light harvesting complex protein of photosystem II) were measured during natural senescence in WT and in two autophagy T-DNA insertion mutants (*atg5* and *atg7*). Thylakoid-localized protein decreased similarly in all genotypes, but stromal protein degradation was incomplete in the two *atg* mutants. In addition, degradation of two stromal proteins was observed in chloroplasts isolated from mid-senescence leaves.

**Conclusions:**

These data suggest that autophagy does contribute to the complete proteolysis of stromal proteins, but does not play a major degenerative role. In addition, support for *in organello* degradation is provided.

## Background

Stromal proteins in C3 mesophyll chloroplasts contain approximately 55% of total cellular nitrogen, mostly in the form of ribulose-1,5-bisphosphate carboxylase/oxygenase (Rubisco), while approximately 20% of total nitrogen is allocated to thylakoid proteins [1]. During senescence most of the nitrogen from these two sources is exported from the aging leaf [2,3], but the proteolytic process is not well understood [4-6]. Genetic approaches towards understanding senescence have focused on the isolation of stay-green mutants, and these studies have shown that stromal and thylakoid proteolysis can be uncoupled. One class of stay-green mutants, nonfunctional type C, retain thylakoid-localized light harvesting complex proteins while stromal proteins are degraded [7,8].

The high nitrogen content of stromal proteins has led to extensive investigation of their proteolysis during leaf senescence. No chloroplast proteases specifically involved in Rubisco or other stromal protein degradation have been identified to date [9]. A Zn-dependent EP1 protease activity was partially purified [10], but no corresponding gene or gene product has been reported. Chloroplast stromal Clp proteases are likely candidates for stromal protein degradation during senescence, however the protein levels of the catalytic ClpP subunit were observed to be greatly diminished in older leaves [11].

Active oxygen treatment led to Rubisco cleavage in isolated chloroplasts [12] and in chloroplast lysates [13,14]. These findings suggested that stromal protein degradation could occur within chloroplasts with high levels of free radicals, a likely condition during the later stages of senescence. However, Rubisco degradation begins during the earliest stages of senescence [4] when photosynthesis is still occurring and free radicals are actively scavenged. For this reason, purified, intact chloroplasts were incubated in the dark to determine if stromal protein degradation could occur in the absence of free radical formation. These chloroplasts were re-purified to be certain they remained intact during the incubation period [15] and four stromal proteins were found to be degraded within intact plastids [16]. Thus, numerous lines of evidence suggest that stromal protein degradation can occur within chloroplasts. However, a cysteine protease inhibitor (cystatin) predominantly expressed in tobacco cytosol inhibited Rubisco degradation in older leaves suggesting that stromal protein degradation is occurring outside of the plastid as well [17].

Although chloroplast numbers only decrease slightly during natural senescence [18], whole chloroplast engulfment via autophagy (chlorophagy) has been observed in individually darkened leaves [19]. The dependence on autophagosome formation was demonstrated by the lack of chlorophagy in the Arabidopsis *atg4a4b* double mutant, however, Rubisco protein levels were found to decrease similarly to wild type in individually darkened leaves of *atg4a4b* mutants [19]. Thus the contribution of chlorophagy to total stromal protein degradation is likely minimal. As most chloroplasts remain intact until the final stages of senescence, extra-plastidic pathways specific to the disposal of stroma proteins have been identified. There have been numerous reports of plastid protuberances that contain Rubisco [20-22], and two distinct entities, Rubisco Containing Bodies (RCBs) and Senescence Associated Vacuoles (SAVs), have been identified.

RCBs are 0.5 to 1.5 μm in diameter, cross-react with antibodies to Rubisco LSU, SSU and chloroplast glutamine synthase (GS2), and have multiple membranes [23]. Stromal-targeted GFP lines have been used to detect RCBs within vacuoles of concanamycin-A treated cells in which vacuolar proteolysis has been prevented due to inhibition of vacuolar-H^+^ ATPases [24]. RCBs appear as Rubisco levels decline in the primary leaves of wheat and are not formed in Arabidopsis *atg5* mutants [25]. ATG5 is required for ATG8 lipidation, and *atg5* mutants cannot form autophagosomes [26,27]. A further connection between autophagy and RCBs is the colocalization of stromal-targeted DsRed and GFP-ATG8, the molecule that coats the autophagosome [28,29]. The presence of RCBs is inversely correlated to starch levels [30], but how this correlates to Rubisco levels is not clear. The decline in Rubisco during natural senescence was measured with RBCS-mRFP fusions, and 10% of the transgenic fusion protein degradation was estimated to be autophagy-dependent [31].

SAVs are 0.5 to 0.8 μm in diameter and were first detected by R-6502, a cysteine protease substrate that becomes fluorescent upon cleavage [32]. Senescent-specific SAVs are acidic compartments that stain with Lysotracker Red and harbor SAG12, a senescence-specific cysteine protease. SAV membranes contain vacuolar H^+^-ATPases, and thus SAVs are considered to be vacuolar compartments. SAVs have also been detected in the *atg7* mutant (which is inhibited at a similar phase of autophagosome formation as *atg5*[33]) indicating SAV formation is not dependent on functional autophagy. SAVs purified on sucrose gradients contained stromal proteins, but not thylakoid proteins, and slow degradation of Rubisco LSU was observed in the isolated SAVs [34].

Nitrogen remobilization efficiency (NRE) was measured in three different Arabidopsis autophagy mutants (*atg5*, *atg9* and *atg18*RNAi) by a ^15^N pulse treatment of leaves and then subsequent transfer of ^15^N into seeds during plant growth [35]. NRE was significantly lower in all autophagy mutants suggesting that autophagy does contribute to nitrogen remobilization. In this study, levels of three native stromal proteins were measured during natural senescence in two autophagy mutants, *atg5* and *atg7*, in order to directly assess the contribution of autophagy towards stromal protein degradation. In addition, degradation of stromal proteins was evaluated in chloroplasts isolated from fully-expanded mid-senescent leaves. Our data provide supporting evidence that autophagy does contribute to stromal, but not to thylakoid, protein degradation, and that stromal proteins might be degraded *in organello*.

## Results and discussion

### Stromal protein degradation is incomplete in autophagy mutants

**Figure 1:**
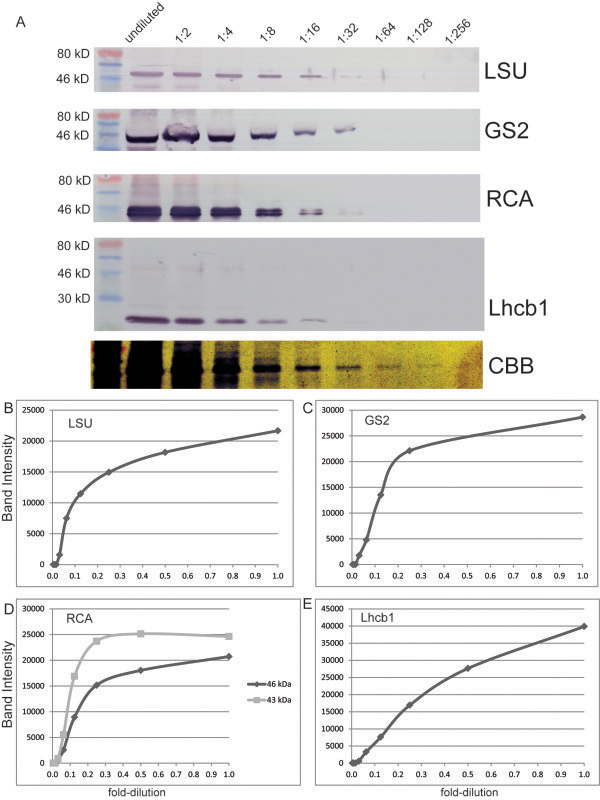
**Antibody detection of two-fold serial dilution of total leaf protein. A**) undiluted and two-fold serial dilutions were detected with the four antibodies used in this study. LSU is Rubisco large subunit, GS2 is chloroplast glutamine synthase, RCA is Rubisco activase, Lhcb1 is the major light harvesting complex protein of photosystem II, CBB is Coomassie Brilliant Blue. **B**-**E**) Band intensity (pixels) was quantified and plotted according to dilution for each of the antibodies as indicated.

Antibodies to three stromal proteins, Rubisco large subunit (α-LSU), glutamine synthase 2 (α-GS2) and Rubisco activase (α-RCA) as well as one thylakoid-localized protein, PSII light harvesting complex protein 1 (α-Lhcb1) were tested against a two-fold serial dilution of total green leaf protein to determine if protein levels could be reliably quantified by immunoblot. Immunoblots and the corresponding quantitation are shown in Figure 1. Proteins recognized by each antibody were found at expected sizes (55 kD for LSU, 42 kD for GS2, 46 kD and 43 kD for RCA and 27 kD for Lhcb1, Figure 1A). Each antibody had its own avidity to its target, for instance α-RCA gives the strongest signal even though LSU is a more abundant protein, yet for all antibodies, signal could be detected at a 1:32 dilution, but not at a 1:64 dilution. Pixel quantitation revealed that α-Lhcb1 was nearly linear (Figure 1E) while the three stromal protein antibodies decreased only slightly for a 1:2 dilution, but demonstrated a steep drop-off at approximately 20% of green leaf protein levels (Figure 1B-D).

**Figure 2:**
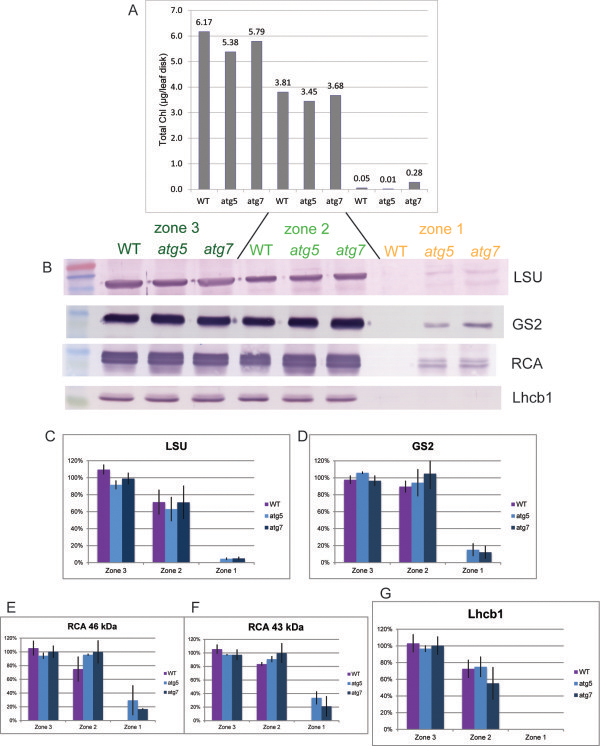
**Stromal and thylakoid protein levels in senescing WT, *atg5 *and *atg7 *. A**) Total chlorophyll levels are shown for zone 3, zone 2 and zone 1 leaf tissue for all three genotypes. All leaves were mature fully-expanded rosette leaves that were fully green (zone 3), partially green (zone 2) or yellow (zone 1). **B**) Immunoblots using the indicated antibodies for zone 3, zone 2, and zone 1 tissue for all three genotypes. **C-G**) Pixels were quantified for three biological replicates of the experiment shown in panel B, and the relative protein abundance in mean values ± standard deviation are shown on the y-axis for each antibody as indicated. The average of zone 3 for all genotypes was set at 100% for each antibody.

To evaluate stromal protein levels in senescing leaves, leaf disks were harvested from mature rosette leaves at different stages of yellowing, and designated as zone 3 (green), zone 2 (yellow-green) and zone 1 (yellow). Chlorophyll per leaf disk was similar for each zone indicating that tissues from different lines were at equivalent stages of chlorophyll loss (Figure 2A). Autophagy mutants become chlorotic at an earlier age than WT due to early activation of the salicylic acid (SA) signaling pathway [26,36,37], so senescent leaf samples were normalized to chlorophyll levels and equal leaf area, and not leaf age. Equal volumes of protein extract from the leaf disks were used in the immunoblots shown in Figure 2B, and band intensity values of three biological replicates are shown in Figures 2C-G. Lhcb1 and chlorophyll levels were similar, as was expected since the light harvesting proteins bind chlorophylls which mutually stabilize one another [38] and are coordinately catabolized [7,39,40]. Only a small change in stromal protein levels was detected between zone 3 and zone 2 for WT and the two autophagy mutants. This is likely a result of non-linearity of antibody binding since chlorophyll and total protein levels have been shown to decrease in parallel in senescing *Lolium temulentum*[8]. Differences between WT and the two autophagy mutants were clearly seen for zone 1 (yellow) tissue in which all three stromal proteins were undetectable for WT, but still detectable for both *atg5* and *atg7*. The retention of the three stromal proteins in yellow tissue of the autophagy mutants suggests that complete degradation of these proteins is autophagy-dependent. Previously, detached leaves from the *atg7* mutant were subject to dark-induced senescence, and Rubisco LSU was found to decrease faster in the *atg7* mutant [33]. However, in this experiment comparisons at similar stages of senescence were not made and the WT samples did not complete senescence since Rubisco LSU levels were still detectable at the last time point. In addition, the molecular process of dark-induced senescence is known to differ from that of natural senescence [41].

Concern exists that the higher stromal protein levels in the younger *atg* tissue resulted from less time for stromal protein degradation and were not related to the loss of autophagy. Double mutants have been constructed between *atg5* and *NahG* as well as *sid2* that decrease SA levels and thus reverse the early senescence phenotype [36]. However the prevention of SA accumulation by *NahG* and *sid2* does increase leaf longevity [41,42] and thus can over-compensate for the early activation of the SA signaling pathway since SA can never accumulate, even at the proper developmental time. Thus an autophagy mutant in a background with normal timing of natural senescence does not yet exist. In addition, if the retention of the three stromal proteins was a result of faster senescence, and not the loss of autophagy, this would indicate that autophagy plays no role in stromal protein degradation, which would be inconsistent with previously published results [31].

### Proteolysis in isolated Arabidopsis chloroplasts

**Figure 3:**
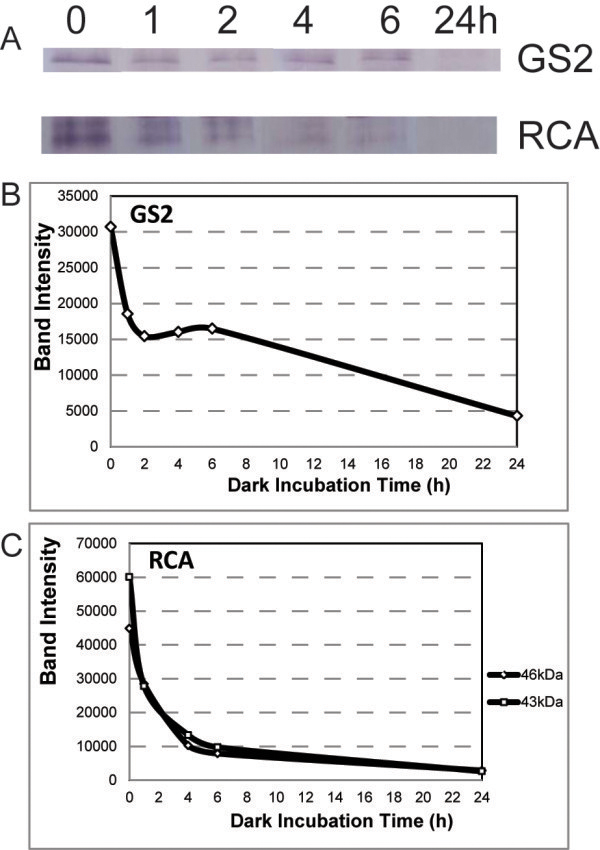
**Degradation of GS2 and RCA within isolated, intact chloroplasts. A**) Chloroplasts were isolated from wild type Arabidopsis leaves and incubated in the dark for the indicated number of hours. Immediately after incubation, intact chloroplasts were purified on a Percoll gradient, and only intact chloroplasts were used for immunoblot analysis with the indicated antibodies. Pixels were quantified for GS2 (**B**) and the two RCA isoforms (**C**).

The substantial decrease in stromal protein levels in the absence of autophagy demonstrates the existence of autophagy-independent proteolytic pathways, and one possibility is within the chloroplast. Previous studies demonstrated stromal protein degradation within isolated, dark-incubated, intact pea chloroplasts [16]. To determine whether stromal protein degradation could occur in chloroplasts isolated from senescent Arabidopsis leaves, a Percoll gradient was used to isolate chloroplasts which were then incubated in the dark, and subsequently purified again by Percoll gradient to insure that only intact chloroplasts were analyzed. Chloroplasts were isolated from mid-senescent (lighter green, yellow tips), mature rosette leaves from 8.5 week old Arabidopsis plants that had large bolts with mature fruit. Figure 3 shows that GS2 and RCA protein levels were greatly diminished after one hour of incubation, but proteolysis continued through the 24 hour incubation period. Surprisingly, Rubisco LSU was not degraded in these intact chloroplasts (data not shown). Similar results were obtained in three independent experiments. Although intact isolated chloroplasts may not mirror *in planta* conditions, the rapid degradation of both RCA and GS2 suggests that stromal proteins can be degraded within chloroplasts isolated from older leaves using a proteolytic mechanism distinct from cytoplasmic autophagy. The stability of Rubisco LSU is likely an artifact of organelle isolation, as it is unlikely that the highly abundant Rubisco is in some way sequestered from stromal proteases.

In an effort to identify chloroplast proteases that might contribute to stromal protein degradation, we isolated T-DNA insertions that disrupted *At5g11650*, a gene encoding a serine protease that is strongly up-regulated in senescent leaves [43]. *At5g11650* is distantly related to pheophytinase [44], but *At5g11650* mutants display normal loss of chlorophyll in older leaves. Stromal protein degradation was identical in chloroplasts isolated from WT and *At5g11650* mutant chloroplasts demonstrating that this chloroplast-localized serine protease is unlikely to play a major role in stromal protein degradation (data not shown).

## Conclusions

Overall, our data suggest that complete degradation of stromal proteins requires autophagy-dependent processes, but much of stromal protein degradation relies on autophagy-independent pathways which may include proteolysis within the chloroplasts or SAVs.

## Methods

### Plant material and growth conditions

Arabidopsis plants were grown under continuous white light (70 μmoles photons m^-2^ sec^-1^) at 24°C in Sunshine Mix #1/LC1 (Sun Gro Horticulture, Inc.) and watered weekly with diluted Gro-Power Liquid (Gro Power, Inc.). SAIL_128_B07 (*atg5-1*, Col-0 ecotype, same allele used in [25]) and SAIL_11_H07 (*atg7*, Col-0 ecotype) were obtained from the Arabidopsis Biological Resource Center (Columbus, OH), and lines homozygous for T-DNA were selected by PCR amplification of genomic DNA.

### Chlorophyll, protein isolation and immunoblots

Two leaf disks (1/4 inch diameter) were incubated in 1.5 ml dimethylformamide for 4–24 hours in the dark at room temperature and total chlorophyll was quantified according to [45]. Protein was extracted from two leaf disks in 133 μL of buffer E [46]. Ten microliters of protein extract were subject to SDS-PAGE (13% acrylamide) and immunoblot analysis [18]. The anti-LSU antibody was generated by Antibodies, Inc. and used at a titer of 1:1,000. Anti-GS2 and anti-Lhcb1 were obtained from Agrisera, Inc. and used at titers of 1:5,000 and 1:10,000, respectively. The anti-RCA antibody was a gift of Dr. Michael Salvucci and used at a titer of 1:5,000. The secondary antibody was goat anti-rabbit coupled to alkaline phosphatase (Millipore, Inc.). Alkaline phosphatase activity was detected by nitroblue tetrazolium and 5-bromo-4-chloro-3’-indolyl phosphate. Blots were scanned and pixels quantified by NIH Image J.

### Chloroplast isolation

The chloroplast isolation protocol was adapted from techniques used in Arabidopsis [47] which was modified from a protocol developed in *Hordeum vulgare*[48]. Additional modifications were adopted from a protocol developed in pea [15,16]. 2.5-5.0 g of mature leaf tissue was minced with a scissors prior to homogenization with a Omni TH tissue homogenizer (Omni, Inc.) in increments of 1.0 to 2.0 g in 30.0 mL Grinding Buffer at 4°C (50.0 mM HEPES-KOH, 2.0 mM EDTA-NaOH, 1.0 mM MnCl_2_, 1.0 mM MgCl_2_, 165.0 mM sorbitol, 5.7 mM ascorbic acid, 0.25% BSA (w/v), final pH 7.5). Non-homogenized tissue was allowed to float to the top while the sample stayed on ice, then only the top 10.0 -15.0 mL was re-homogenized to avoid disturbing existing contents. Homogenate was then filtered through one layer of Miracloth in increments of 5.0 mL, clearing debris from the Miracloth in between addition of more homogenate. Filtered homogenate was then centrifuged at 1000 × g for 8 minutes at 4°C.

The resulting pellet was resuspended in 4.0 mL of Grinding Buffer, and loaded onto a 40-85% Percoll step gradient in a 15.0 mL centrifuge tube loaded with 4.0 mL 85% solution and 3.0 mL 40% solution [40% solution: 40.0% Percoll (GE Healthcare Bio-Sciences), 330 mM sorbitol, 2.1 mM MgCl_2_, 1.6 mM MgCl_2_, 50 mM HEPES-KOH pH 7.6, 2.0 mM EDTA-NaOH pH 8.0, 0.1% (w/v) BSA); 85% solution: 85.0% Percoll, 50 mM HEPES-KOH pH 7.6, 330 mM sorbitol]. 40-85% Percoll step gradients containing the resuspended chloroplasts were centrifuged at 6,000 × g for 15 minutes at 4°C. Intact chloroplasts were collected from the 85% solution surface, washed with 30.0 mL of Incubation Buffer [50.0 mM HEPES-KOH, 1.0 mM MgCl_2_, 1.0 mM MgCl_2_, 165.0 mM sorbitol, 5.7 mM ascorbic acid, 0.25% BSA( w/v), final pH 7.5], and centrifuged at 1,000 × g for 6 minutes.

The chloroplast pellet was resuspended in 1.0 mL of Incubation Buffer, and chlorophyll concentration was adjusted to 200 μg/mL. Chloroplasts were incubated in a foil-wrapped Oakridge tube to prevent light exposure, and stored in a closed drawer at room temperature. Harvested samples were immediately loaded onto a 40-85% Percoll gradient and centrifuged for 15 minutes at 6,000 × g. Intact chloroplasts were collected from the 85% solution surface, washed in 30.0 mL of Incubation Buffer and centrifuged at 1000 × g for 6 minutes. The resulting pellet was then resuspended in Incubation Buffer and stored at −80°C for immunoblot analysis.

## Abbreviations

ATG: Autophagy; CBB: Coomassie Brilliant Blue; GS2: Chloroplast glutamine synthetase; kD: KiloDalton; Lhcb1: Major light harvesting complex proteins of photosystem II; LSU: Large subunit; NRE: Nitrogen remobilization efficiency; RCA: Rubisco activase; RCB: Rubisco Containing Body; Rubisco: Ribulose bisphosphate-1,5-carboxylase/oxygenase; SA: Salicylic acid; SAV: Senescence associated vacuole.

## Competing interests

The authors declare that they have no competing interests.

## Authors’ contributions

TL designed and carried out the *atg* studies, SVW designed and carried out the isolated chloroplast studies, and JB designed experiments and drafted the manuscript. All authors read and approved the final manuscript.
